# Role of information in consumers’ preferences for eco-sustainable genetic improvements in plant breeding

**DOI:** 10.1371/journal.pone.0255130

**Published:** 2021-07-29

**Authors:** Massimiliano Borrello, Luigi Cembalo, Riccardo Vecchio

**Affiliations:** Department of Agricultural Sciences, University of Naples Federico II, Napoli, Italy; Groupe ESC Dijon Bourgogne, FRANCE

## Abstract

Consumers’ preferences for products derived from genetic improvements and innovations in plant breeding are often conditioned by technophobia and negative public imaginaries. The current study addresses this issue by analyzing consumers’ monetary preferences for a win-win innovation (generating gains for both private actors and the community) in the viticulture sector, namely fungus resistant grapes (FRG). The use of these grapes reduces the quantity of chemical inputs applied to vineyards, simultaneously improving firms’ economic performance. This study aimed to assess whether consumers prefer wines originating from FRG varieties to conventional wines. In particular, through an experimental online survey involving 627 Italian regular wine drinkers, the study compares individuals’ willingness to pay (WTP) for conventional wines with the WTP for two FRG wines produced with two different techniques: horticultural hybridization and genome editing. The study also assesses the potential effect of polarized media coverage on preferences by testing, in a between-subjects experimental design, two diverging (positive/negative) information scenarios, and the core drivers of these preferences. The findings suggest that respondents express a premium price for horticultural FRG wines compared to conventional wines (+9.14%) and a strong discount for genome edited FRG wines (–21.13%). The results also reveal that negative information reduces consumers’ WTP for horticultural FRG wines, while positive information increases their WTP for genome edited FRG wines. Last, the study highlights that individuals concerned with food sustainability issues and knowledgeable about wine are more likely to accept both FRG typologies. Overall, the study confirms the crucial role of appropriate information for market acceptance of innovations based on plant genetics to foster the adoption of sustainable pest-reducing practices in wine production.

## 1. Introduction

Transitioning toward sustainable agri-food systems is a theme of ever-growing relevance [1]. However, while in principle the need for this transition is well accepted, based on the current discourse, sustainable agri-food systems shape different futures/paradigms in which agro-ecological practices including localized/regionalized agriculture are opposed to agro-industrial production, often referred to as hypermodern and highly technological production alternatives [2–5]. These two perspectives are divisive and polarize the debate on agricultural sustainability. On one hand, the adoption of ecological principles in farming production has been blamed for reducing yields, being unable to guarantee farmers’ revenue, and utopist in the context of increasing global food demand [6]. On the other, the agro-industrial paradigm is criticized for reproducing highly contested intensive production schemes, thus being inappropriate in approaching sustainable planetary boundaries and natural equilibriums [7]. As for the latter approach, sustainable agricultural intensification [8] has been charged with over-reliance on the adoption of genetic engineering to increase productivity while facing environmental problems [9], and public opinion has strongly shaped the trajectories of agricultural biotechnologies [10]. However, new insights into plant genetics and biotechnology advancements are still of paramount importance for international agendas concerning sustainable agriculture. Specifically, genetic improvements of crops through conventional breeding [11, 12] and new breeding technologies [13] are currently adopted to generate plant hybrids to cope with biotic (e.g., pests) and abiotic (e.g., drought) stressors.

Nevertheless, effective innovations may result in poor acceptance rates due to information that may jeopardize their diffusion. This is the case for innovations that generate tensions based on ideological positions. One example is the acceptance of genetically modified organisms (GMOs), which have been contrasted, particularly in the EU [4]. To examine whether and how polarized information influences the acceptance of innovations in plant genetics, the current research focused on a specific agricultural sector, namely viticulture, in which novel vine varieties able to face major fungal diseases (e.g., downy and powdery mildews and gray rot) have captured the interest of many practitioners. Specifically, fungus resistant grape (FRG) varieties are vine hybrids that require fewer chemical inputs, offering a solution to one of the main concerns of viticulture, namely the environmental impacts and toxicity associated with exposure to synthetic pesticides [14]. Although pesticide risks are a longstanding issue for industrialized agricultural systems, the persistence of synthetic chemical compounds in the environment and food still engages international institutions, such as the 50% pesticide reduction target by 2030 of the European Green Deal Farm to Fork Strategy, and the European Food Safety Authority (EFSA) report on pesticide residues in food [15]. Regarding viticulture, the data in the EFSA report demonstrates the wide use of pesticides (more than 86% of grapes analyzed in the report had quantifiable pesticide residues). In recent times, this problem has stimulated the uptake of wines labeled as organic [16], biodynamic [17], or natural [18], all of which rely on a still controversial treatment-oriented strategy. These alternative production approaches mostly aim to countervail the environmental impacts of synthetic pesticides used in conventional viticulture to treat vineyards against cryptogamic diseases as well as other diseases and insect pests [19]. However, these approaches also have shortcomings in terms of environmental impacts. For example, sulphur- and copper-based formulations used by organic viticulture to replace conventional inputs, such as the Bordeaux mixture [20], can accumulate in the environment at dangerous levels [21]. Consistently, from February 1, 2019, a decision of the European Commission stipulates that winegrowers reduce the copper used in vineyards to 28 kg/Ha in the following 7 years (Commission Implementing Regulation (EU) 2018/1981, [22]). Therefore, approaching fungal diseases by replacing the conventional grapevine species (*i*.*e*., *Vitis vinifera*) with fungus resistant grape varieties is an alternative for disease prevention [23]. FRG varieties prevent cryptogamic diseases while reducing the use of chemical inputs, thus improving economic and environmental sustainability. Noteworthy is that this provides a potential reduction of more than 80% in chemical treatments [24], far above the 50% reduction target imposed by the European Green Deal. FRG varieties can be developed through two main approaches: horticultural interspecific crossing and genome editing. FRG generated with the first approach (hereafter *horticultural hybrids*) were first issued by crossbreeding *V*. *vinifera* and North American and Asian *Vitis* species carrying high fungal resistant proprieties and non-*V*. *vinifera* genes, resulting in interspecific hybrids [25–28]. More recent developments of this approach have generated FRG varieties that maintain a high percentage of the *V*. *vinifera* genome (up to 99%) [29, 30], thus preserving the original organoleptic properties. FRG generated with the second approach (hereafter *genome edited hybrids*) take advantage of new DNA sequencing methods able to map relevant regions of the plant genome [31] and associated with the adoption of targeted genetic scissors (*e*.*g*., the Nobel Prize awarded technology CRISPR-Cas9 [32]). While several studies have implemented genome editing to grapevines [see among others, 33–40], the technological readiness level of genome edited hybrids is still low, and they are not yet commercially available. However, because of its ability to implement targeted and highly precise modifications to plant genes, genome editing might be a game changer in the wine sector, with even higher potential than horticultural hybrids [30]. In summary, considerably reducing the amount of chemical inputs through highly elaborate breeding programs and a low impact on the original genetic pool of grapevines, FRG has a pivotal role in the sustainability of the future wine sector. Since FRG simultaneously improves firms’ economic performance, these innovations can be considered a win-win solution for viticulture as they can generate both economic gains for private actors and environmental benefits for the community [41, 42].

The current research focuses on the market acceptance of new plant breeding systems for sustainable viticulture, specifically on consumers’ preferences for FRG varieties. In particular, consumer preferences are detected in different information scenarios that depict polarized/ideological positions related to these innovations.

In addition to limited insights [43, 44], consumers’ views of FRG remain unexplored. The existence of market segments interested in sustainable wine is well documented, with a recent review stating that “there is a considerable segment of consumers across different countries with positive perceptions pertaining to sustainable production methods of wine, who are willing to pay a premium for such a wine” [45, p. 388]. While this this might be a driving force for the FRG wine market, consumers’ acceptance of sustainable wines (e.g., organic ones) [46] cannot be transposed *sic et simpliciter* to FRG wines, whose future success should not be taken for granted. Even if new hybrids can preserve most of the original *V*. *vinifera* pedigree, technologies used to induce resistance might be perceived as a source of sophistication compromising wine quality in terms of oenological and traditional attributes [30]. Furthermore, similar to other technologies implemented in food production, technophobic individuals might be concerned and therefore reject horticultural interspecific crossing and genome editing applied to grapevines [47]. Indeed, appropriate information that can make consumers aware of FRG technological specificities might have a crucial role in determining future acceptance or negative misperceptions of FRG wines. As Lusk et al. [48, p. 82] stated, “The technical differences between different breeding techniques are likely beyond comprehension for most consumers.” In a study by McFadden and Lusk [49], when asked about differences between some genetic modification techniques, most consumers responded, “I don’t know.” To exacerbate this baseline, there is evidence that in the past, public perception of innovations in plant biotechnology has been conditioned by the construction of negative imaginaries (*e*.*g*., the case of GMOs) [4, 10, 50]. Communication about FRG is forced to rebrand genetic agricultural innovations to cope with this backdrop. If this is not the case, the risk is that the uptake of FRG will mirror the path of past advancements in plant genetics, where misguided messages perpetuate negative beliefs in public opinion but scientific evidence hardly enters the debate.

Based on these considerations, this study investigates the issue of consumers’ monetary preferences for FRG wines by comparing individuals’ willingness to pay (WTP) for FRG wines and their WTP for conventional wines. Based on an experimental survey (the study protocol was registered on Aspredicted in November 2020) involving 627 Italian wine drinkers, we sought to address the following four research questions:

*RQ1*. Do consumers prefer FRG wines to conventional ones?
*RQ2*. Do consumers have different preferences for FRG wines generated from horticultural hybrids compared to FRG wines generated from genome edited hybrids?
*RQ3*. To what extent does information (positive or negative) affect consumers’ preferences for FRG wines?
*RQ4*. What are the determinants of consumers’ preferences for FRG wines?

FRG wines have a disruptive potential to contribute to the environmental sustainability of wine production and powerfully decrease vineyard operating costs [28]. However, hybridization technologies related to wine are at the dawn of their development, and wine businesses need specialized market knowledge to achieve the successful commercial exploitation of FRG wines [30].

## 2. Materials and methods

### 2.1. Experimental procedure

An online between-subject experiment involving three experimental groups was conducted using the *SurveyMonkey*® platform. The platform randomly assigned respondents to the information treatment conditions. In total, 275 completed responses were collected for the control group and 176 for the treatment groups. Experiment participants expressed their preferences for three wine typologies: conventional wine, FRG wine produced with horticultural hybrids, and FRG wines produced with genome edited hybrids. No mention was made of other product characteristics (*e*.*g*., red vs. white wine, vine variety, origin).

**Fig 1:**
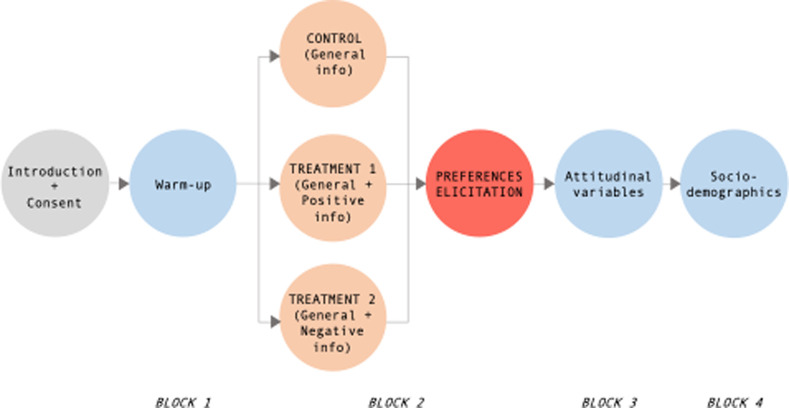
Experimental procedure.

After a short introduction, participants signed the informed consent. The ethical review and approval were waived for this study because of the observational nature of the research (consumer data provided on a voluntary basis) and full compliance of the study with the principles stated in the Declaration of Helsinki. Then, participants responded to a questionnaire composed of eight sections (S1 File) divided into four building blocks (Fig 1 reports the full experimental procedure). In the first block (sections 1 and 2), participants were asked to provide warm-up preliminary information concerning their wine consumption and wine purchasing habits. The second block (section 3) served to collect information on their monetary preferences for FRG wines. Specifically, to elicit individual WTP for conventional and FRG wines, participants were shown (in randomized order) three 0.75 L mock wine bottles carrying information on the wine typologies and the EU mandatory labeling information. Subsequently, respondents stated the maximum amount of money they were willing to pay for the three bottles selecting among an array of ordered prices ranging from €1 to €16, with 1€ intervals. To limit the hypothetical bias of WTP elicitation, a consequentiality script [51] was adopted before participants stated their monetary preferences. In the third block (sections 4–7), information on a set of attitudinal variables was measured. Finally, the fourth block included questions concerning participants’ socio-demographic characteristics (section 8).

### 2.2. Information on treatment conditions

We elaborated two information scenarios built around the FRG environmental sustainability aspects and potential consumers’ food technophobia. More specifically, we assumed that future media coverage might strongly influence consumers’ acceptance of FRG wines. As elaborated previously, FRG wines might be perceived as highly positive (environmentally friendly) or highly negative (representing genetic manipulation). Even for horticultural hybrids, which are not the result of genetic engineering, misleading heuristics might occur depending on how the media and wine sector convey information on FRG origin. The assumption that simplistic and polarized information conveyed by the media might be detrimental in transferring to lay people correct scientific knowledge on FRG rests on evidence drawn from past innovations in plant genetics, particularly genetically modified crops [4, 52–55]. Based on this, we posit that positive (negative) information about FRG increases (decreases) consumers’ preference for FRG wines and decreases (increases) consumers’ preference for conventional wine.

Before running the experimental survey, the information treatments were tested to ensure they were actually perceived by respondents as intended. Therefore, two consecutive preliminary studies were performed asking individuals to indicate their assessment of three types of information—control, a “positive information” treatment, and “negative information” treatment—on a nine-point anchored scale. The findings of the first pre-study (*n* = 30) led to adjustments in both positive and negative treatments based on poorly significant differences in the mean evaluations. The results of the second pre-study (*n* = 50) confirmed that after the changes in wording and pictures, the three information conditions were assessed by wine consumers in a significantly different (neutral, negative, and positive) manner.

After these two pre-studies, the experimental survey was structured as a between-subject design. Participants were randomly assigned to one of three groups differing in the amount and type of information provided (*i*.*e*., control, “positive information” treatment, “negative information” treatment). The control group, serving as a reference, was provided with only general information about the grapes used for producing conventional wine, FRG wine generated from horticultural hybrids, and FRG wines generated from genome edited hybrids. This control group represents the preferences of wine drinkers without the influence of media coverage as posited in the current study. The information scripts were as follows.

- Conventional: *These grapes are those used for producing wine you usually buy at the retail store*.
- Horticultural hybrids: *These grapes are derived from crossbreeding between conventional grapes and other grape species undertaken in the field with horticultural techniques*. *They are varieties of conventional grapes whose DNA includes genes from other grape species*. *These varieties are not GMOs*.
- Genome edited hybrids: *These grapes are derived from advanced laboratory techniques (genome editing) that allow modification of the DNA of conventional grapevines more precisely than the techniques of genetic manipulation used in the past*, *thus reducing potential undesired DNA alterations*. *These varieties are GMOs*.

Before eliciting their preferences, the participants of the two treatment groups received additional information concerning the wine typologies (full information condition). This information was not provided as a replacement for the general information provided to the control group, but as a complement. In line with previous approaches in food and wine consumption studies [e.g., 56, 57], two short newspaper-like articles were created to communicate different opinions about FRG wines. While one article focused on the positive aspects of FRG regarding the sustainability of winemaking and human health, the second focused on possible negative aspects related to hybridization and contamination of the wine. Each article consisted of a headline, an image, and the main text. Both articles were created by adapting the format of existing international press articles. Moreover, two pictures were selected, one for each article, and used as a background to strengthen the effect of positive/negative information [58]. This strategy is consistent with previous findings showing the use of visuals to influence public perceptions of genetic technologies [59, 60]. Respondents in the two treatment groups read their respective articles immediately before stating their maximum WTP for the three wine typologies.

### 2.3. Sample characteristics

A random convenience sample of 627 Italian individuals aged more than 18 years who consumed wine more than once a year participated in the experiment. Subjects were recruited by means of social networks and word of mouth. The sample is not representative of the national population of regular wine drinkers or the general Italian population. Compared to statistics on national regular wine drinkers (2019 data from the Multipurpose Survey of Daily Life of the Italian Statistics Institute, ISTAT [61]), the final sample here includes a slightly higher share of female individuals (+3%), a strong over-representation of highly educated respondents (54% vs. 14% with a university degree or higher), and a lower number of daily consumers (11.8% vs. 17.6%). Furthermore, considering the general Italian population aged more than 18 years, the survey respondents in this study heavily under-represented retired, unemployed, and housewife individuals, while including a very high share of young adults, students, and citizens living in the southern area of the country. Therefore, the interpretation of the results must consider these important shortcomings of the convenience sample.

Table 1 provides the descriptive statistics of the complete sample.

**Table 1:** Socio-demographic characteristics of the sample (*N* = 627).

		%	
*Gender*	Female	47.2%	
	Male	52.8%	
*Age*	Number of years–mean (S.D.)	32.9	(11.7)
*Household size*	Number of components–mean (S.D.)	3.52	(1.29)
*Education level*			
	Primary	0.2%	
	Secondary	1.7%	
	High	44%	
	University	40.7%	
	Post-graduate	13.4%	
*Employment*			
	Employee	40.2%	
	Freelance	17.2%	
	Student	33.2%	
	Housewife	1.9%	
	Retired	1.8%	
	Unemployed	5.7%	
*Household average monthly income*	<2000 €	40%	
	2000 €–4000 €	37.8%	
	> 4000 €	22.2%	
*Geographic origin*			
	North	16.3%	
	Centre	15.1%	
	South and islands	68.6%	

As for the wine habits of the sampled individuals, 70.2% of respondents declared they drink wine at least once a week; their preferred wine consumption and purchase locations were respectively, their own home (54.4%) and large retailers (43.2%). Furthermore, 46.1% of respondents stated they spent on average between 3 € and 10 € for a bottle of wine. Table 2 shows the detailed statistics on participants’ wine habits.

**Table 2:** Wine habits of the sample (*N* = 627).

		%
**Wine consumption frequency**	Daily	11.8
	4–5 times a week	9.4
	2–3 times a week	24.4
	Once a week	24.6
	2–3 times a month	12
	Once a month	6.7
	More than once a year	11.2
**Preferred wine consumption location**	At home	54.4
	At my friends/relatives’ house	20.4
	At the restaurant	11.3
	At wine bars	13.4
	Other	0.5
**Most frequent wine purchase site**	Supermarket, hypermarket, discount	43.2
	Direct purchase from wine producers	21.5
	Wine shop	23
	Online	7.8
	Other	4.5
**Average price paid for a 0.75 L bottle (proxy = price paid for the last bottle of wine purchased for dinner with relatives/friends)**	< 3 €	3.7
	3–6 €	21.4
	6–10 €	24.7
	10–15 €	20.1
	15–20 €	16.7
	> 20 €	13.4

### 2.4. Metrics applied in the experiment

To explain individuals’ preferences, diverse attitudinal variables were collected using a set of metrics consistent with the experimental design. Specifically, the following four validated scales were employed, which measured information on a seven-point scale anchored at the extremes and in the middle: the wine subjective knowledge scale [adapted from 62], wine involvement scale [adapted from 63], abbreviated food technology neophobia scale [64], and sustainability concerns scale [65]. The literature shows that subjective knowledge and wine involvement are powerful predictors of wine choices [66, 67]. The wine subjective knowledge scale used in the current research includes items such as “I feel quite knowledgeable about wine” and “Compared to most other people, I know less about wine.” The wine involvement scale includes items such as “I have a strong interest in wine” and “I would choose my wine very carefully.” The third scale was included to assess the effect on WTP of individuals’ attitudes toward novel technologies applied to food. It includes items such as “The benefits of new food technologies are often grossly overstated” and “Society should not depend heavily on technologies to solve its food problems.” As the FRG discourse is strongly aimed at highlighting the positive impact on the sustainability of winemaking, the fourth selected scale measures individuals’ concerns about sustainability issues in food production. It considers various aspects of food sustainability, measuring for instance, the level of concern for “The use of pesticides used in food production” and “Poor working conditions and wages for food producers.” All internal consistencies of the constructs described above exceeded a reliable threshold value. In addition to the validated scales, to test the potential influence of negative attitudes on participants’ concern and opposition toward GMOs, they were asked to respond to two items from Fernbach and colleagues [68]. The two items, measured on a seven-point scale anchored at the extremes and in the middle, are as follows: “Please indicate your level of concern about genetically modified foods” and “Please indicate your level of opposition to genetically modified foods.” Table 3 reports the means, standard deviations, and Cronbach’s α of the metrics.

**Table 3:** Mean scores of applied metrics.

Scales	Mean	S.D.	α Cronbach’s
**Wine subjective knowledge**	4.10	1.67	0.86
**Wine involvement**	5.05	1.46	0.94
**Sustainability concerns**	5.73	1.05	0.94
**Abbreviated food technology neophobia**	3.99	1.11	0.87
**Negative attitudes towards GMOs**	4.77	1.65	-

*Notes*: Anchoring for Wine subjective knowledge, Wine involvement, Food healthiness, and Abbreviated Food Technology Neophobia were: 1—totally disagree and 7—totally agree. For sustainability concerns, the anchoring was: 1—only slightly concerned and 7—extremely concerned. 4 expressed neutrality for all metrics.

### 2.5. Empirical model

For the estimation of the role of selected factors driving consumers’ preferences for FRG wines, a seemingly unrelated regression model (SUR) must be adopted. The SUR is a multivariate linear regression model suited to contexts where the estimation of a system of equations is needed [69, 70]. In this case, the model consists of two linear regression equations: one for each FRG typology (hh = horticultural hybrids; geh = genome-edited hybrids). More formally, the following equations were estimated for the *i*-th respondent:

$$\{\begin{array}{l} \Delta WTP_{hh,i}=x^{\prime }\beta_{hh}+e_{hh,i}\\ \Delta WTP_{geh,i}=x^{\prime }\beta_{geh}+e_{geh,i} \end{array}$$

The two dependent variables ΔWTP represent the differences in WTP between wines originating from conventional wine and grapevine hybrids, *x* is a vector of explanatory variables, and the error terms *e* are assumed independent across individuals and correlated across equations. In each equation, the estimate of the statistically significant coefficient *β* identifies and measures the corresponding determinants of consumer preferences (measured as WTP). The SUR model was estimated with a common set of socio-demographic variables in each indicator, while attitudinal variables varied across equations. Statistical and graphical elaborations were performed using STATA v.16.

## 3. Results

### 3.1 Consumer preferences for FRG wines

**Fig 2:**
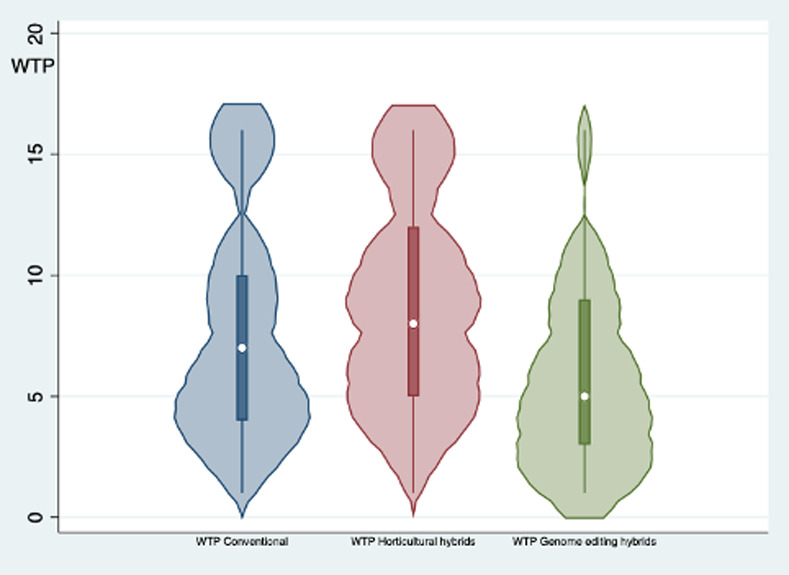
Violin plot of WTP (€) for the three wine typologies (*N* = 627) reporting boxplots and estimated Kernel density.

**Fig 3:**
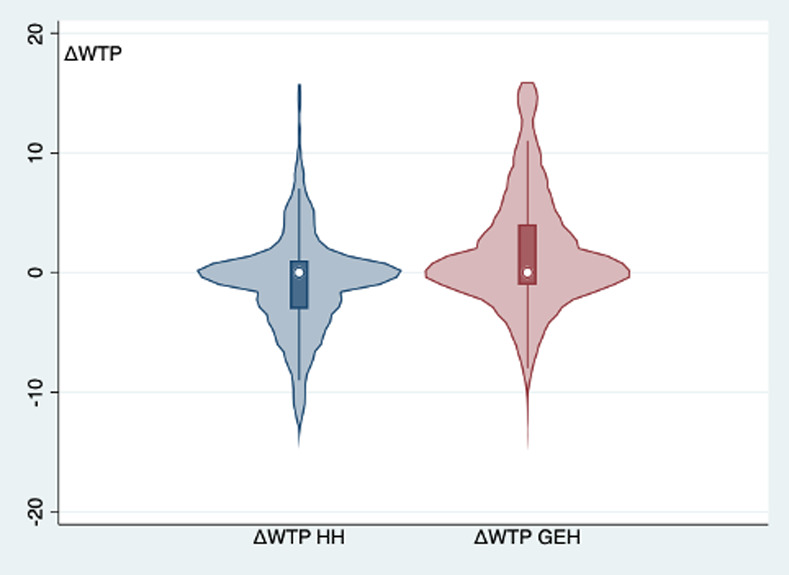
Violin plot of ΔWTP (€) (*N* = 627) reporting boxplots and estimated Kernel density. ΔWTP_HH_ = WTPc−WTP_hh_; ΔWTP_GEH_ = WTP_c_—WTP_geh_.

To uncover consumers’ preferences for FRG wines and explore whether there is a difference between preferences for FRG wines generated from horticultural hybrids and those for FRG wines generated from genome edited hybrids (RQ1 and RQ2), the WTP distributions were analyzed via parametric and non-parametric tests. Fig 2 depicts the distribution of respondents’ WTP for conventional wine, FRG wine produced with horticultural hybrids, and FRG wine produced with genome edited hybrids. The distributions of the three WTPs reveal that consumers prefer FRG wine produced with horticultural hybrids to conventional wines, which in turn, are preferred to FRG wines generated from genome edited hybrids (Fig 2). In particular, considering the full sample (627 individuals), the average WTP for horticultural hybrids is 8.75 € (S.D. 4.6), for conventional wines 7.95 € (S.D. 4.2), and for genome edited hybrids 6.27 € (S.D. 4.3). According to the pairwise comparisons (via a *t*-test and Wilcoxon signed rank test), the differences between the measured means and WTP distributions of the three wine typologies are all statistically significant. In particular, the resulting premium price for horticultural hybrids amounts to 0.8 € (S.D. 4.3), while the price discount for genome edited hybrids is 1.68 € (S.D. 4.9). In percentage terms, ΔWTP between conventional wine and the two FRG wines amounts to +9.14% (for horticultural hybrids) and -21.13% for (genome edited hybrids). Fig 3 shows the distributions of the differences in WTP, indicating that the ΔWTP for genome edited hybrids is characterized by a more heterogeneous and diverse response from consumers.

### 3.2 Information effect and preference drivers

To assess the effects of different information (positive and negative) on consumers’ preferences for FRG wines and to identify the determinants of individuals’ WTP for these wines (RQ3 and RQ4), a SUR model was applied. Table 4 shows that different information treatments have diverse effects on preferences for the two FRG wines. Specifically, belonging to the negative treatment group increased the probability of having low differential WTP between horticultural hybrids and conventional wines (negative effect on preferences for horticultural hybrids), whereas belonging to the positive treatment group increased the probability of having low differential WTP between conventional wines and genome edited hybrids (a positive effect on preferences for genome edited hybrids).

**Table 4:** Seemingly unrelated regression coefficients.

Equations	Obs	Parms	RMSE^^^		R-square	
**ΔWTP**_**HH**_	627	9	4.146		0.117	
**ΔWTP**_**GEH**_	627	9	4.551		0.239	
			**ΔWTP**_**HH**_		**ΔWTP**_**GEH**_	
**Wine subjective knowledge**			-0.360	***	-0.531	***
**Wine involvement**			0.025		0.110	
**Sustainability concerns**			-0.600	***	-0.468	***
**Negative attitude toward GMOs**			-0.007		0.446	***
**Abbreviated Food Technology Neophobia**			0.076		0.353	*
**Age**			0.058	***	0.049	***
**Education**			0.462	**	0.304	
**Positive information**			-0.269		-1.246	***
**Negative information**			0.723	*	0.440	

*Notes*: Dependent variables: ΔWTP_HH_ = WTPc−WTP_hh_; ΔWTP_GEH_ = WTP_c_—WTP_geh_.

^^:^ Root Mean Square Error.

Asterisks represent statistical significance at the following levels: * *p*≤0.1,

** *p*≤0.05,

*** *p*≤0.01. Breusch-Pagan test of independence: Chi-square 265.678***.

In addition, the econometric model enabled us to predict the influence of certain variables and scales on consumer preferences. Preferences for FRG wines produced with horticultural hybrids are positively influenced by wine subjective knowledge and concerns for the sustainability of food (*i*.*e*., an increase in the level of knowledge and sustainability concerns leads to higher preferences for horticultural hybrids). However, preferences for FRG are negatively influenced by age and education. Regarding the information effect on the WTP for horticultural hybrids, the model shows that negative messages decrease preferences, while positive communication is not statistically significant.

Considering preferences for FRG wines produced with genome edited hybrids, the SUR estimates show that preferences for these wines are positively influenced by wine subjective knowledge and sustainability concerns, and they are negatively influenced by food technology neophobia and concerns about the safety of GMOs. For socio-demographics, age decreases preferences (i.e., older respondents express lower WTP), while the number of household members positively affects the WTP for these wines.

## 4. Discussion

While consumers in developed countries are increasingly advocating for more sustainable agri-food systems [71], these requests are not always sided by an unconditional acceptance of agricultural biotechnology applications [72]. Indeed, public opinion has forcefully influenced the adoption of agricultural biotechnologies in the recent past [10]. Thus, understanding the drivers and barriers of public perceptions of these advancements is paramount to inform policymakers interested in fostering pest-reducing practices.

The present study investigated consumers’ monetary preferences for FRG wines by comparing individuals’ WTP for FRG wines produced with horticultural and genome edited hybrids with their WTP for conventional wines. Furthermore, it assessed the effect of polarized information on participants’ preferences and the core drivers of individual preferences.

As previous research demonstrated, technological advancements applied in the food domain can receive diverse levels of consumer approval [73, 74]. The current study also suggests that consumers have positive preferences for FRG wines produced with horticultural hybrids, but do not support FRG wines generated from genome edited hybrids. This outcome is likely connected to consumers’ perception of horticultural hybrid FRG wines as products that can occur in nature [75, 76] and do not involve gene modifications [77]. In addition, individuals have negative perceptions of foods they consider unnatural [78]. The findings also prove that providing consumers with different types of information can affect their preferences for these wines, corroborating previous studies that demonstrated that positive messages enhance preferences [79, 80] while negative imaginaries decrease them [50]. This is also consistent with previous findings showing that providing detailed information on wines produced with genetic technologies can improve consumers’ perception of these wines [81]. Analyzing respondents’ WTP for FRG wines through a SUR model, several individual characteristics were found to effectively drive preferences. Sustainability concerns play a key role in fostering support for both horticultural hybrids and genome edited FRG wines, confirming the importance of this feature in the modern wine market [45]. Similarly, higher levels of wine subjective knowledge increased preferences for FRG wines, confirming that diverse levels of product knowledge translate into different consumer segments [82]. In particular, the current findings suggest that individuals perceived to be more well-informed on wine are less skeptical of FRG wines, as they probably rely more on other product attributes to form their preferences [83]. Age and education also have a negative influence on preferences for FRG wines. Older individuals are generally less prone to innovations, particularly when they refer to traditional products such as wine. As for education, previous knowledge of other sustainable alternatives in winemaking may have influenced the preferences of more educated individuals. The future of FRG wines depends largely on how final consumers perceive these products and how the media will frame this information. A wine process that does not recall GM food and is strongly promoted as an important contribution to increasing sustainability is likely to be well accepted by consumers. Therefore, FRG from horticultural hybrids appears to be a much more promising venture, which consumers are already likely to accept and eventually, even be willing to pay a premium price for. Policymakers and wine practitioners should thus carefully consider consumers’ perceptions and personal characteristics when communicating about FRG wines, avoiding potentially confusing messages and the generation of misleading, detrimental heuristics [84].

The current study is subject to several caveats. First, the application of a random, convenience sample limits the extension of results to the general Italian wine consumer population (and even more to different cultural contexts). Second, the measurement of individuals’ stated preferences (WTP collected through an online survey) is prone to hypothetical bias [85] and social desirability bias [86]. Furthermore, self-selection bias [87] of the sample could have affected the final outcomes of the study, which over-represented individuals generally more favorable to FRG wines. Finally, the study might have overlooked other important drivers of consumer preferences (e.g., wine sensory characteristics).

## 5. Conclusions

The European Green Deal Farm to Fork strategy targets a 50% reduction of pesticides in the European Union agricultural sector by 2030. New breeding techniques and genetic improvements of crops present great opportunities to achieve this goal and allow farmers to powerfully reduce operating costs. However, market acceptance of products resulting from these technologies is not assured.

The current study explored consumers’ preferences for FRG wines produced with horticultural hybrids and genome edited hybrids. The findings suggested that individuals express a willingness to pay a premium price for the former FRG compared to conventional wines and a discount for the latter FRG wines. This study also explored the effect of different types of information on individual preferences to better understand the potential impact of polarized messages on consumer behavior. Because of the limited number of available studies, individuals’ preferences regarding FRG wines should be further explored by investigating other research questions applying different types of preference elicitation techniques. Undoubtedly, FRG varieties will be a core theme in the sustainable transformation of future agri-food systems.

## Supporting information

S1 File Translated full survey. This Word file contains the questionnaire submitted to participants through the *SurveyMonkey*® platform. After the introduction, the file is divided in the eight sections described in the experimental procedure section (2.1). In Section 3, it also includes pictures of the three 0.75 L mock wine bottles and of the two short newspaper-like articles showed to treated participants.
(DOCX)
