# Supplementary material for: Role of information in consumers’ preferences for eco-sustainable genetic improvements in plant breeding
Source: PLoS One. 2021 Jul 29;16(7):e0255130. doi: 10.1371/journal.pone.0255130 (PMC8321114; doi:10.1371/journal.pone.0255130)
Supplement: S1 File — This Word file contains the questionnaire submitted to participants through the SurveyMonkey® platform. After the introduction, the file is divided in the eight sections described in the experimental procedure section (2.1). In Section 3, it also includes pictures of the three 0.75 L mock wine bottles and of the two short newspaper-like articles showed to treated participants. (DOCX) [file pone.0255130.s001.docx]

**Questionnaire translated from Italian**

Thank you for participating to this survey on wine consumption carried out by the University of Naples Federico II.

This survey has no commercial purposes. The questionnaire is anonymous, information will be analyzed in an aggregated way and results will be used only for scientific purposes by the University of Naples Federico II. The questionnaire fully complies with the principles stated in the Declaration of Helsinki concerning ethical principles in research.

Filling in the questionnaire will require about 12 minutes. There are no right or wrong answers, we are only interested in your opinion. The topic of the survey is wine consumption.

Section 1

Please, respond to a couple of questions concerning your consumption habits related to wine.

*How frequently do you drink wine?*

|  | Everyday |
| --- | --- |
|  | 4-5 times a week |
|  | 2-3 times a week |
|  | Once a week |
|  | 2-3 times a month |
|  | Once a month |
|  | More than once a year |
|  | Never |

*Where do you drink wine more frequently?*

|  | At home |
| --- | --- |
|  | At my friends/relatives’ house |
|  | At the restaurant |
|  | At wine bars |
|  | Other (please, specify) |

Section 2

Please, respond to some question concerning your purchase habits related to wine.

*Where do you purchase wine more frequently?*

|  | Supermarket, hypermarket, discount |
| --- | --- |
|  | Direct purchase from wine producers |
|  | Wine shop |
|  | Online |
|  | Other (please, specify) |

*What is the price of the last bottle of wine you have purchased for a dinner with friends and/or relatives?*

|  | < 3 € |
| --- | --- |
|  | 3-6 € |
|  | 6-10 € |
|  | 10-15 € |
|  | 15-20 € |
|  | 20-50 € |
|  | > 50 € |

Section 3

(Control, positive treatment and negative treatment groups answered to different versions of this section)

We are going to introduce you two innovative technique in winemaking.

The wine we usually drink is produced with conventional grapes. By crossbreeding conventional wine and other grape species, recent experiments have produced new types of hybrid grapes. Hybrid grapes can be produced by means of two different techniques: in the field (horticultural hybrids) or in laboratories (genome edited hybrids).

Please, read the following newspaper article that provides you more information on hybrid grapes (only for positive treatment and negative treatment groups).

| Positive treatment | Negative treatment |
| --- | --- |
| 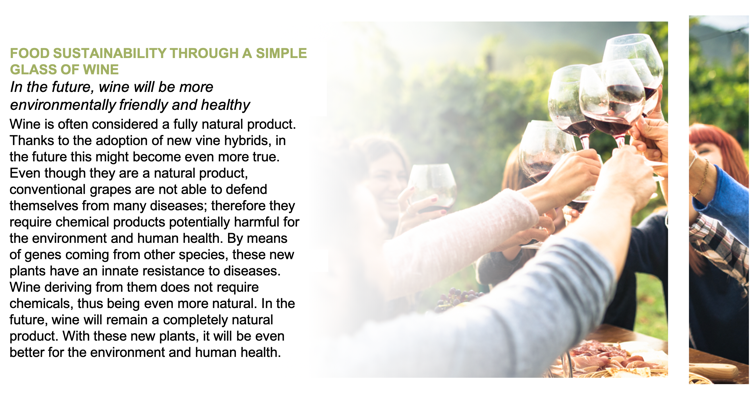 | 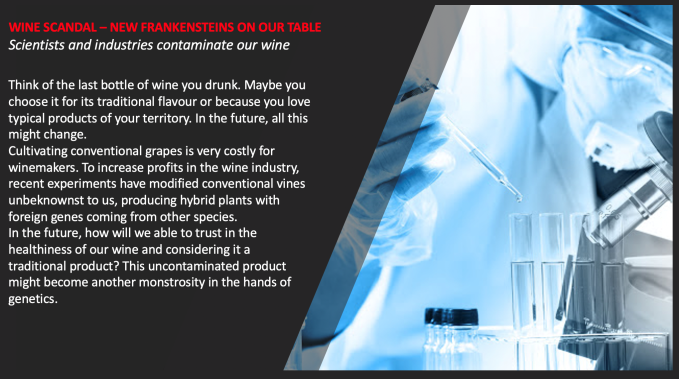 |

Please, refer to the price you would pay for a wine bottle to drink at home with your friends and/or relatives; consider also that the average price for a red wine bottle, at the supermarket, is 3,70€. *What is your maximum willingness to pay for a wine bottle produced with conventional and hybrid grapes?*

According to recent studies, sometimes individuals give some responses, but then they behave differently in real life. Please, when declaring your willingness to pay, respond exactly as if you were in a real shop.

*What is your maximum willingness to pay for a 0,75 L wine bottle produced with …*

| *… conventional grapes?*  These grapes are those used for producing the wine you usually buy at the supermarket. | | | | | | | | | | | | | | | |
| --- | --- | --- | --- | --- | --- | --- | --- | --- | --- | --- | --- | --- | --- | --- | --- |
| 1 € | 2 € | 3 € | 4 € | 5 € | 6 € | 7 € | 8 € | 9 € | 10 € | 11 € | 12 € | 13 € | 14 € | 15 € | 16 € |
|  |  |  |  |  |  |  |  |  |  |  |  |  |  |  |  |

*What is your maximum willingness to pay for a 0,75 L wine bottle produced with …*

| *… horticultural hybrid grapes?*  These grapes are derived from crossbreeding between conventional grapes and other grape species undertaken in the field with horticultural techniques. They are varieties of conventional grapes whose DNA includes genes from other grape species. These varieties are not GMOs. | | | | | | | | | | | | | | | |
| --- | --- | --- | --- | --- | --- | --- | --- | --- | --- | --- | --- | --- | --- | --- | --- |
| 1 € | 2 € | 3 € | 4 € | 5 € | 6 € | 7 € | 8 € | 9 € | 10 € | 11 € | 12 € | 13 € | 14 € | 15 € | 16 € |
|  |  |  |  |  |  |  |  |  |  |  |  |  |  |  |  |

*What is your maximum willingness to pay for a 0,75 L wine bottle produced with …*

| *… genome edited hybrid grapes?*  These grapes are derived from advanced laboratory techniques (genome editing) that allow modification of the DNA of conventional grapevines more precisely than the techniques of genetic manipulation used in the past, thus reducing potential undesired DNA alterations. These varieties are GMOs. | | | | | | | | | | | | | | | |
| --- | --- | --- | --- | --- | --- | --- | --- | --- | --- | --- | --- | --- | --- | --- | --- |
| 1 € | 2 € | 3 € | 4 € | 5 € | 6 € | 7 € | 8 € | 9 € | 10 € | 11 € | 12 € | 13 € | 14 € | 15 € | 16 € |
|  |  |  |  |  |  |  |  |  |  |  |  |  |  |  |  |

Section 4

We have passed the middle of the questionnaire. Please, respond now to some question on your wine knowledge.

*Please, indicate your level of agreement with the following opinions, using a scale from 1 to 7 (1 = totally disagree; 4 = neutral; 7 = totally agree).*

|  | 1 | 2 | 3 | 4 | 5 | 6 | 7 |
| --- | --- | --- | --- | --- | --- | --- | --- |
| I feel quite knowledgeable about wine |  |  |  |  |  |  |  |
| I do not feel very knowledgeable about wine |  |  |  |  |  |  |  |
| Among my friends, I am one of the “experts” on wine |  |  |  |  |  |  |  |
| Compared to most other people, I know less about wine |  |  |  |  |  |  |  |
| When it comes to wine, I really do not know a lot |  |  |  |  |  |  |  |

Please, respond now to some question on your wine interest.

*Please, indicate your level of agreement with the following opinions, using a scale from 1 to 7 (1 = totally disagree; 4 = neutral; 7 = totally agree).*

|  | 1 | 2 | 3 | 4 | 5 | 6 | 7 |
| --- | --- | --- | --- | --- | --- | --- | --- |
| I have a strong interest in wine |  |  |  |  |  |  |  |
| Wine is very important to me |  |  |  |  |  |  |  |
| For me, wine do matter |  |  |  |  |  |  |  |
| I would choose my wine very carefully |  |  |  |  |  |  |  |
| Deciding which wine to buy would be an important decision for me |  |  |  |  |  |  |  |
| Which wine I buy matters to me a lot |  |  |  |  |  |  |  |

Section 5

Could you please give us some information on your opinion about new technologies applied to food?

*Please, indicate your level of agreement with the following opinions, using a scale from 1 to 7 (1 = totally disagree; 4 = neutral; 7 = totally agree).*

|  | 1 | 2 | 3 | 4 | 5 | 6 | 7 |
| --- | --- | --- | --- | --- | --- | --- | --- |
| New foods are not healthier than traditional foods |  |  |  |  |  |  |  |
| The benefits of new food technologies are often grossly overstated |  |  |  |  |  |  |  |
| There are plenty of tasty foods around so we do not need to use  new food technologies to produce more |  |  |  |  |  |  |  |
| New food technologies decrease the natural quality of food |  |  |  |  |  |  |  |
| New food technologies are unlikely to have long term negative health effects |  |  |  |  |  |  |  |
| New food technologies may have long term negative environmental effects |  |  |  |  |  |  |  |
| It can be risky to switch to new food technologies too quickly |  |  |  |  |  |  |  |
| Society should not depend heavily on technologies to solve its  food problems |  |  |  |  |  |  |  |
| There is no sense trying out high-tech food products because the  ones I eat are already good enough |  |  |  |  |  |  |  |

Section 6

Could you please give us some information on your opinion about sustainability in food production?

*Please, indicate your level of concern for the following issues, using a scale from 1 to 7 (1 = only slightly concerned; 4 = neutral; 7 = extremely concerned).*

|  | 1 | 2 | 3 | 4 | 5 | 6 | 7 |
| --- | --- | --- | --- | --- | --- | --- | --- |
| The use of child labour in food production |  |  |  |  |  |  |  |
| Deforestation of the rain forest |  |  |  |  |  |  |  |
| Starvation and malnutrition in the world population |  |  |  |  |  |  |  |
| The use of pesticides used in food production |  |  |  |  |  |  |  |
| Poor treatment of animals in food production |  |  |  |  |  |  |  |
| Environmental damage caused by human use of land and water |  |  |  |  |  |  |  |
| The amount of food that is wasted |  |  |  |  |  |  |  |
| Using too much of the world’s natural resources for food production |  |  |  |  |  |  |  |
| Poor working conditions and wages for food producers |  |  |  |  |  |  |  |
| Packaging that is not recyclable |  |  |  |  |  |  |  |
| The amount of packaging used on products |  |  |  |  |  |  |  |
| Carbon emissions caused by food production |  |  |  |  |  |  |  |
| The amount of energy used when transporting food products |  |  |  |  |  |  |  |
| The amount of energy used when cooking food products |  |  |  |  |  |  |  |

Section 7

Before we move to the last section of the questionnaire, could you please give us some information on your opinion about genetically modified organisms (GMO)?

*Please, answer to the following questions using a scale from 1 to 7.*

|  | 1 | 2 | 3 | 4 | 5 | 6 | 7 |
| --- | --- | --- | --- | --- | --- | --- | --- |
| *Please indicate your level of concern about genetically modified foods (1 = not concerned at all; 4 = neutral; 7 = extremely concerned)* |  |  |  |  |  |  |  |

|  | 1 | 2 | 3 | 4 | 5 | 6 | 7 |
| --- | --- | --- | --- | --- | --- | --- | --- |
| *Please indicate your level of opposition to genetically modified foods (1 = not opposed at all; 4 = neutral; 7 = extremely opposed)* |  |  |  |  |  |  |  |

Section 8

The survey is almost over. Could you please respond to the following questions on your socio-demographic characteristics?

*Gender*

|  | Male |
| --- | --- |
|  | Female |
|  | Other |

*Age*

Drop-down list 18-100

*Number of family members*

Open-ended answer

*Education level*

|  | Primary school |
| --- | --- |
|  | Secondary school |
|  | High school |
|  | University |
|  | Post-graduate education (e.g., PhD) |

*Employment*

|  | Employee |
| --- | --- |
|  | Free lance |
|  | Student |
|  | Housewife |
|  | Retired |
|  | Unemployed |

*Household income*

|  | < 2000 € |
| --- | --- |
|  | 2000-4000 € |
|  | > 4000 € |

*Province of residence*

Drop-down list with provinces abbreviations

*Do you live in a wine production area?*

|  | Yes |
| --- | --- |
|  | No |
